# Increased recurrent risk did not improve cerebrovascular disease survivors’ response to stroke in China: a cross-sectional, community-based study

**DOI:** 10.1186/s12883-020-01724-1

**Published:** 2020-04-21

**Authors:** Shengde Li, Li-Ying Cui, Craig Anderson, Chunpeng Gao, Chengdong Yu, Guangliang Shan, Longde Wang, Bin Peng, on behalf of the FAST-RIGHT Investigators and Coordinators

**Affiliations:** 1 Department of Neurology, Peking Union Medical College Hospital, Peking Union Medical College and Chinese Academy of Medical Sciences, Shuaifuyuan1, Dong Cheng District, Beijing, 100730 China; 2 Neurological and Mental Health Division, The George Institute for Global Health, Faculty of Medicine, University of New South Wales, Sydney, Australia; 3 The George Institute for Global Health, Peking University Health Science Center, Beijing, China; 4 Disease Control and Prevention Office, Dalian Municipal Central Hospital, Dalian, Liaoning China; 5 Department of Epidemiology and Statistics, Institute of Basic Medical Sciences, Chinese Academy of Medical Sciences, Beijing, China; 6 Stroke Control Project Committee, The National Health Commission, Beijing, China

**Keywords:** Health knowledge, attitudes, practice, Awareness, Emergency medical services, Cerebrovascular disease, Survivors, Risk factors

## Abstract

**Background:**

Cerebrovascular disease (CVD) survivors are at a high risk of recurrent stroke. Although it is thought that survivors with higher risk of stroke respond better to stroke onset, to date, no study has been able to demonstrate that. Thus, we investigated whether the intent to call emergency medical services (EMS) increased with recurrent stroke risk among CVD survivors.

**Methods:**

A cross-sectional community-based survey was conducted from January 2017 to May 2017, including 187,723 adults (age ≥ 40 years) across 69 administrative areas in China. A CVD survivor population of 6290 was analyzed. According to the stroke risk score based on Essen Stroke Risk Score, CVD survivors were divided into three subgroups: low (0), middle (1–3) and high (4–7) recurrent risk groups. Multivariable logistic regression models were used to identify the association between the stroke risk and stroke recognition, as well as stroke risk and EMS calling.

**Results:**

The estimated stroke recognition rate in CVD survivors with low, middle, and high risk was 89.0% (503/565), 85.2% (3841/4509), and 82.5% (1001/1213), respectively, while the rate of calling EMS was 66.7% (377/565), 64.3% (2897/4509), and 69.3% (840/1213), respectively. The CVD survivors’ knowledge of recognizing stroke and intent to call EMS did not improve with recurrent stroke risk, even after adjustment for multiple socio-demographic factors.

**Conclusions:**

Despite being at a higher risk of recurrent stroke, Chinese CVD survivors showed poor knowledge of stroke, and their intent to call EMS did not increase with recurrent stroke risk. Enhanced and stroke risk-orientated education on stroke recognition and proper response is needed for all CVD survivors.

## Background

In China, as in many countries, stroke is a major health challenge, with an increasing number of stroke-related deaths and disabilities [1–4]. Reperfusion therapy with intravenous alteplase, can improve the outcomes of acute ischemic stroke (AIS). However, less than 2% of AIS Chinese patients receive alteplase primarily due to pre-hospital delays [5, 6]. Emergency medical services (EMS) usage provides quick access to hospitals, however, a very low proportion of AIS patients use EMS in China [6–9]. Survivors of CVD are at higher risk of severe stroke [10–13]. In China, common vascular risk factors such as hypertension, diabetes and dyslipidemia are not well adequately managed [14–16], and many of them are oblivious to their stroke risk factors [17]. Thus, EMS seems more important to patients with higher recurrent risk of stroke. However, previous study reported that a significant ratio of 34.6% of Chinese CVD survivors did not call EMS [18]. The details on stroke recognition and responses among CVD survivors remained unclear. Therefore, based on the FAST-RIGHT study, we aimed to determine the characteristics of CVD survivors, and whether the intent to call EMS increased with the recurrent stroke risk.

## Methods

Data were obtained from the FAST-RIGHT study, part of the China National Stroke Screening Survey (CNSSS), and included 69 administrative areas between January 2017 and May 2017. More details on the CNSSS are outlined elsewhere [18, 19], and can also be found on the website of the National Health Commission [20]. The CNSSS was a cross-sectional community-based survey with a 2-stage stratified sampling framework based on county-level demographic data. In the FAST-RIGHT study, residents aged 40 years and older were screened by trained research staff using a standard face-to-face questionnaire regarding sociodemographic, medical, and family history, lifestyle factors, and four specific questions regarding stroke awareness (See Supplementary Appendix 1, Additional file 1). Commencement of stroke education was also recommended after completing the questionnaire survey. All screening data were transferred from questionnaires to an electronic database and checked centrally for completeness and errors by an experienced data manager. The FAST-RIGHT study was approved by the central ethics committee of Peking Union Medical College Hospital (the principal study center), and all participants provided written informed consent.

### Explanatory and outcome variables

Recognition of stroke symptoms was defined as a participant’s unprovoked awareness of “facial droop,” “arm weakness,” and “speech disturbances” (slurred speech, or word-finding difficulties) [21]. Calling EMS immediately after the onset of any of these symptoms was regarded as the correct action in response to stroke. A reported history of stroke was confirmed by a neurologist or physician. The modifiable risk factors included hypertension, diabetes, dyslipidemia, overweight and obesity (BMI = 24–50), atrial fibrillation (AF)/valvular heart disease/coronary heart disease/other heart disease, smoking (including current, former, and passive smoking), drinking (including current and former drinking), and limited exercise (See Supplementary Appendix 2, Additional file 1). Survey participants were divided into two subgroups: CVD survivors and non-CVD population. CVD survivors were defined as those with a history of cerebrovascular disease, including ischemic stroke, cerebral hemorrhage, subarachnoid hemorrhage, and transient ischemic attacks. Individuals without a history of cerebral vascular disease were classified as the non-CVD.

### Assessment of recurrent stroke risk

Based on the Essen Stroke Risk Score (ESRS), a stroke risk score was developed with more scores indicating higher stroke risk, including age, hypertension, diabetes, smoking and heart disease. The stroke risk score was divided as three levels: low risk (0), middle risk (1–3) and high risk (4–7) [22, 23] (See Supplementary Appendix 3, Additional file 1).

### Statistical analyses

The stroke recognition rate (SRR) and correct action rate (CAR) of total, low-, middle-, and high-risk groups were determined based on the specific subgroups, which was defined by age, sex, site (urban vs. rural), region, education level, and annual income among CVD survivors. The association between the increased recurrent stroke risk and stroke recognition, as well as calling EMS were analyzed using multivariable logistic regression.

Based on stroke risk score, we compared the socio-demographic factors among the low-, middle- and high-risk groups among CVD survivors. A standard two-sided *P* value (< 0.05) was considered statistically significant. All analyses were performed using SAS version 9.3.

## Results

Of the 187,723 residents screened for eligibility, 6290 were CVD survivors and were included in our analysis (See Supplementary Table S1 and Figure S1, Additional file 1). Stroke risk score showed a U-shaped association with CAR, but negative correlation with SRR. In the low-, middle- and high-risk subgroups, the SRRs were 89.0, 85.2, and 82.5%, respectively (*P* = 0.0014), while CARs were 66.7, 64.3, and 69.3%, respectively (*P* = 0.004) (See Supplementary Table S2, Additional file 1). In total group and low-, middle- and high-risk subgroups, the estimated SRR and CAR varied across regions and socioeconomic statuses (Table 1). Across most different subgroups, CAR was 5.3–40.0% lower than SRR. Among those who recognized the onset of a stroke, only 67.9% intended to call EMS (See Supplementary Table S3, Additional file 1).

**Table 1:** SRR and CAR by demographic, socio-economic, and stroke risk variables in CVD survivors

	Case, N	SSR, n (%)				CAR, n (%)			
		0	1–3	4–7	Total	0	1–3	4–7	Total
Age (years)									
40–49	301	91 (87.5)^aa^	163 (86.2)^cc^	6 (75.0)^bb^	260 (86.4)^cc^	71 (68.3)	122 (64.6)^cc^	7 (87.5)	200 (66.5)^bb^
50–59	1039	252 (92.3)	630 (88.5)	39 (72.2)	921 (88.6)	192 (70.3)	490 (68.8)	41 (75.9)	723 (69.59)
60–69	2376	160 (85.1)	1624 (87.1)	283 (88.4)	2070 (87.1)	114 (60.6)	1220 (65.4)	225 (70.3)	1560 (65.66)
70–79	1943	..	1128 (82.7)	476 (82.2)	1604 (82.6)	..	849 (62.2)	400 (69.1)	1249 (64.28)
80–99	631	..	296 (78.1)	197 (78.2)	493 (78.1)	..	216 (57.0)	167 (66.3)	383 (60.70)
Sex									
Male	2938	150 (88.2)	1832 (84.8)	511 (84.5)	2496 (85.0)	104 (61.2)	1394 (64.5)	431 (71.2)	1930 (65.7)
Female	3352	353 (89.4)	2009 (85.5)	490 (80.6)	2852 (85.1)	273 (69.1)	1503 (64.0)	409 (67.3)	2185 (65.2)
Site									
Urban	2932	185 (92.0)	1724 (86.6)^aa^	625 (84.7)^aa^	2537 (86.5)^bb^	159 (79.1)^cc^	1470 (73.9)^cc^	565 (76.6)^cc^	2195 (74.9)^cc^
Rural	3358	318 (87.4)	2117 (84.0)	376 (79.2)	2811 (83.7)	218 (59.9)	1427 (56.6)	275 (57.9)	1920 (57.2)
Regions									
North + Northeast	718	54 (98.2)^aa^	445 (96.5)^cc^	185 (91.6)^cc^	684 (95.3)^cc^	32 (58.2)^cc^	299 (64.9)^cc^	159 (78.7)^cc^	490 (68.3)^cc^
East	1510	106 (81.5)	933 (85.8)	252 (86.3)	1291 (85.5)	56 (43.1)	594 (54.6)	198 (67.8)	848 (56.2)
Central	2726	224 (90.0)	1692 (85.0)	390 (80.1)	2306 (84.6)	195 (78.3)	1396 (70.2)	342 (70.2)	1933 (70.9)
South	392	24 (92.3)	252 (81.6)	39 (69.6)	316 (80.6)	19 (73.1)	178 (57.6)	34 (60.7)	232 (59.2)
Southwest	622	59 (88.1)	365 (79.2)	77 (81.9)	501 (80.6)	46 (68.7)	331 (71.8)	68 (72.3)	445 (71.5)
Northwest	322	36 (94.7)	154 (77.0)	58 (70.7)	250 (77.6)	29 (76.3)	99 (49.5)	39 (47.6)	167 (51.9)
Education									
≤ Primary	3247	200 (86.6)	1947 (80.8)^cc^	477 (78.8)^bb^	2624 (80.8)^cc^	122 (52.8)^cc^	1368 (56.7)^cc^	370 (61.2)^cc^	1860 (57.3)^cc^
Middle/High school	2733	276 (90.8)	1700 (90.1)	463 (85.9)	2442 (89.4)	231 (76.0)	1367 (72.4)	416 (77.2)	2015 (73.7)
≥ College	310	27 (90.0)	194 (91.9)	61 (88.4)	282 (91.0)	24 (80.0)	162 (76.8)	54 (78.3)	240 (77.4)
Personal Annual Income (US $)									
< 731	2479	214 (88.4)	1500 (81.9)^cc^	315 (77.8)^cc^	2029 (81.9)^cc^	140 (57.9)^cc^	1002 (54.7)^cc^	252 (62.2)^cc^	1394 (56.2)^cc^
731–2923	2005	172 (86.4)	1218 (82.9)	264 (79.0)	1656 (82.6)	134 (67.3)	1003 (68.2)	228 (68.3)	1365 (68.1)
> 2923	1803	117 (94.4)	1120 (93.0)	422 (89.0)	1660 (92.1)	103 (83.1)	892 (74.1)	360 (75.9)	1356 (75.2)
Living Status^a^									
With family	5849	489 (89.2)	3593 (85.3)	897 (82.7)	4982 (85.2)	364 (66.4)	2690 (63.8)^aa^	747 (68.9)	3802 (65.0)^bb^
With others	438	14 (82.4)	245 (83.9)	104 (80.6)	363 (82.9)	13 (76.5)	207 (70.9)	93 (72.1)	313 (71.5)
Children number									
0	57	2 (100.0)	36 (83.7)^cc^	9 (75.0)^cc^	47 (82.5)^cc^	2 (100.0)^cc^	33 (76.7)^cc^	9 (75.0)^cc^	44 (77.2)^cc^
1	1228	157 (91.8)	790 (91.2)	174 (91.6)	1122 (91.4)	140 (81.9)	641 (74.0)	151 (79.5)	932 (75.9)
2–3	3876	306 (87.9)	2394 (85.6)	608 (83.3)	3309 (85.4)	213 (61.2)	1767 (63.2)	514 (70.4)	2494 (64.3)
≥ 4	1124	37 (86.0)	617 (77.2)	210 (74.7)	865 (77.0)	21 (48.8)	455 (56.9)	166 (59.1)	643 (57.2)
Awareness of stroke									
No	942	..	..	..	..	26 (41.9)^cc^	343 (51.3)^cc^	113 (53.3)^cc^	482 (51.2)^cc^
Yes	5348	..	..	..	..	351 (69.8)	2554 (66.5)	727 (72.6)	3633 (67.9)
Avenues^b^									
1	3103	221 (84.0)^bb^	1802 (79.9)^cc^	431 (73.7)^cc^	2455 (79.1)^cc^	166 (63.1)^aa^	1433 (63.6)^cc^	388 (66.3)	1987 (64.0)^cc^
2–3	2936	243 (92.7)	1875 (89.7)	526 (90.4)	2646 (90.1)	178 (67.9)	1324 (63.3)	416 (71.5)	1919 (65.4)
4–6	251	39 (97.5)	164 (99.4)	44 (95.7)	247 (98.4)	33 (82.5)	140 (84.8)	36 (78.3)	209 (83.3)

*SRR* Stroke recognition rate, *CAR* Correct action rate

Stroke risk score, its range was 0 to 7 and was divided as three subgroups: 0, 1–3, and 4–7

n: number for recognizing stroke/correct action to stroke in each cell; The total number of each cell was shown in Supplementary Table S4 of Additional file 1

^a^With family includes living with spouse/children; With others includes being single, living in a nursing home, and with other people

^b^Number of avenues taken to learn about acute stroke

^aa^*P* value < 0.05

^bb^*P* value < 0.01

^cc^*P* value < 0.001

Those without superscripts in the first line of every item: *P* value ≥0.05

**Fig. 1:**
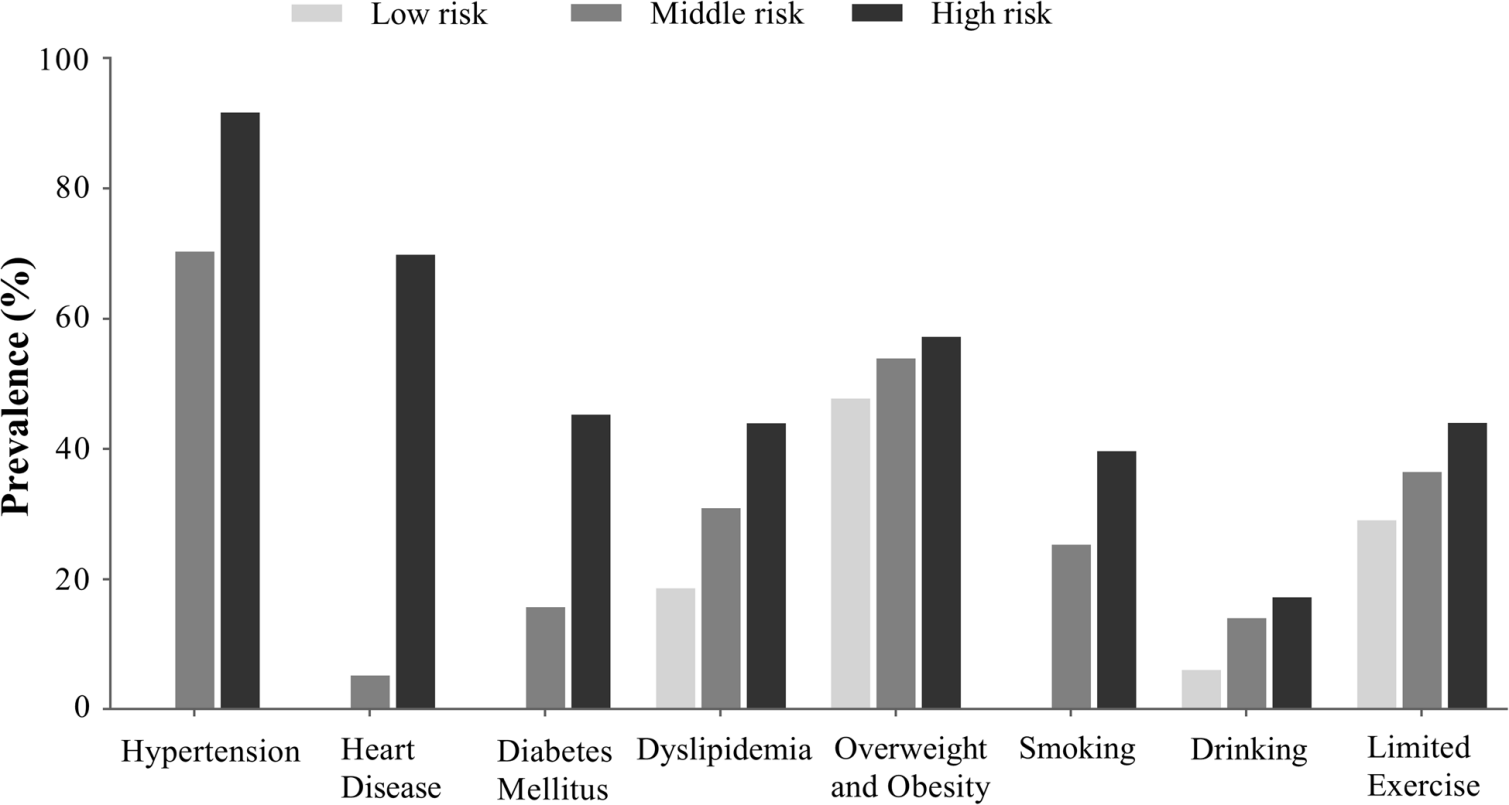
Prevalence of modifiable cardiovascular risk factors by stroke risk score. Low risk: 0 for stroke risk score. Middle risk: 1–3 for stroke risk score. High risk: 4–7 for stroke risk score. Heart disease includes atrial fibrillation (AF), valvular heart disease, coronary heart disease and other heart disease. Smoking includes current, former and passive smoking. Drinking includes current and former drinking. Overweight and obesity indicates body mass index:24–50

The high-risk subgroup was older, and had higher education and income levels (See Supplementary Table S4, Additional file 1). The proportions of family history of stroke (33.6%) and urban adults (60.8%) were significantly higher in high-risk subgroup. Compared with low-risk subgroup, middle- and high-risk CVD survivors had markedly higher prevalence of modifiable cardiovascular risk factors (Fig. 1).

**Fig. 2:**
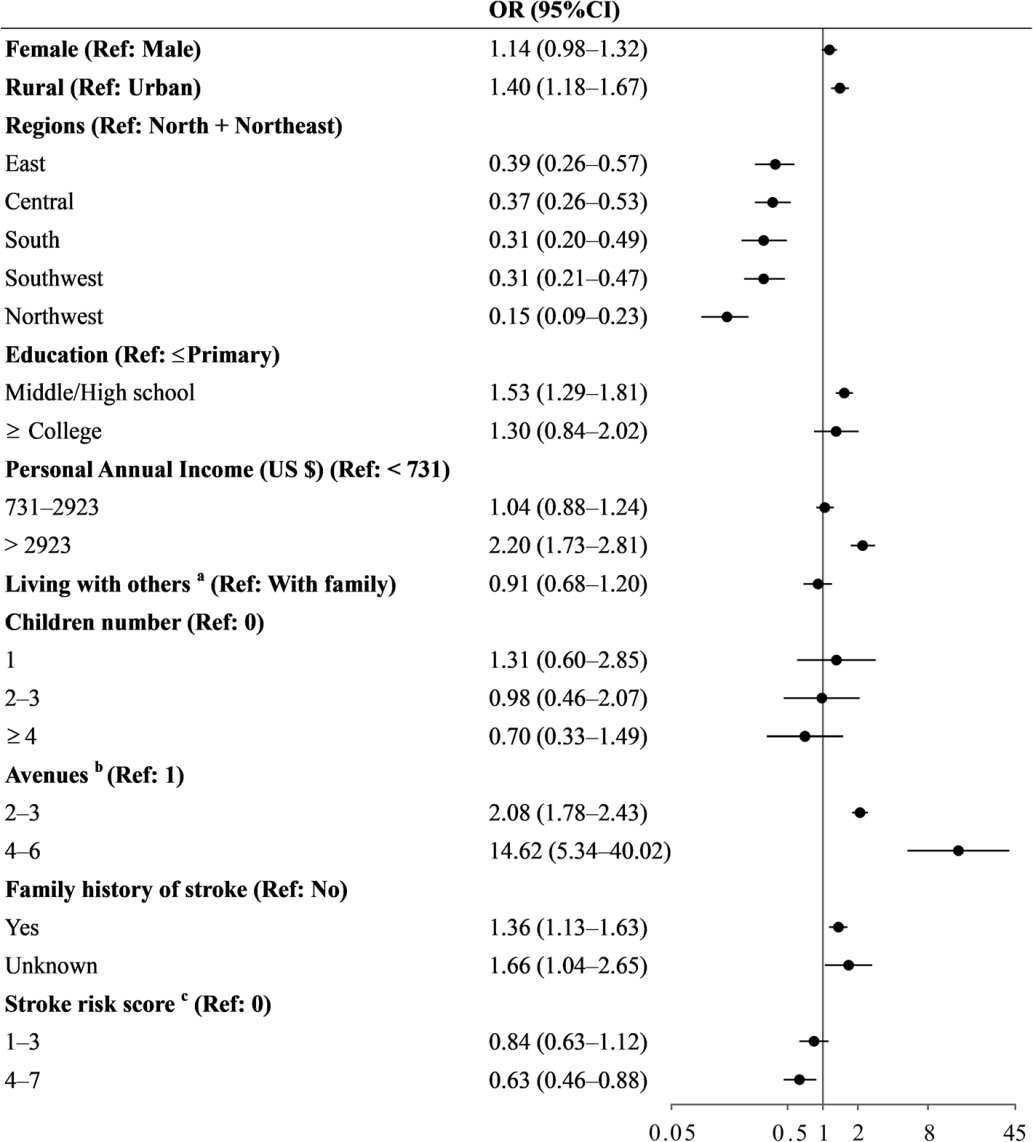
Logistic regression model of factors associated with stroke recognition. ^a^ With family includes living with spouse/children; With others includes being single, living in a nursing home, and with other people. ^b^ Number of avenues taken to learn about acute stroke. ^c^ Stroke risk score range was 0 to 7

**Fig. 3:**
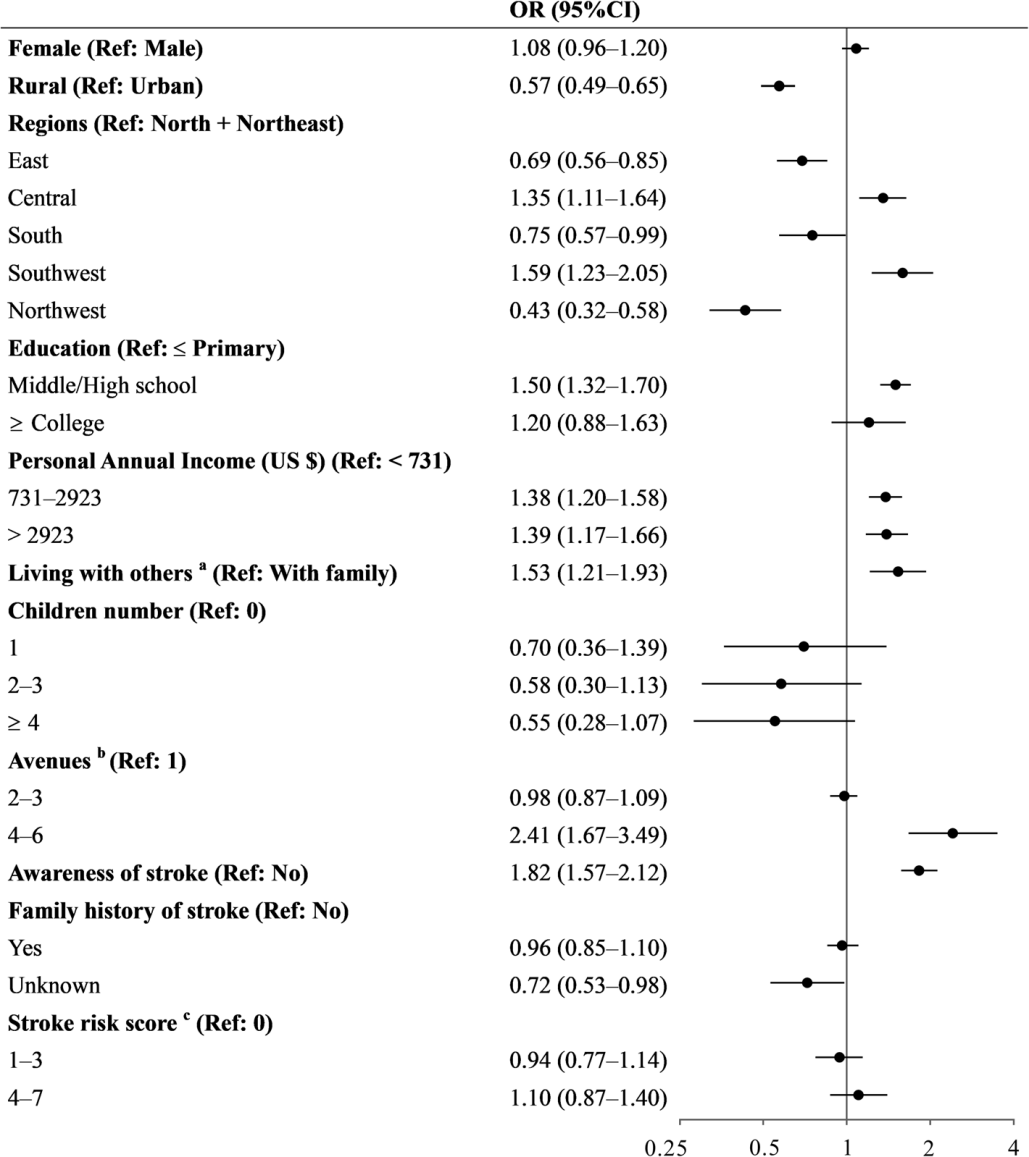
Logistic regression model of factors associated with calling EMS. ^a^ With family includes living with spouse/children; With others includes being single, living in a nursing home, and with other people. ^b^ Number of avenues taken to learn about acute stroke. ^c^ Stroke risk score range was 0 to 7

Figures 2 and 3 show the variables independently associated with recognition of stroke symptoms and calling EMS, respectively. The CVD survivors’ ability of stroke recognition and intent to call EMS did not improve with increasing stroke risk score. Moreover, out of the multiple factors, only urban-rural sites, region, education, annual income, living status, stroke awareness, number of avenues to learn about stroke, and family history were statistically significant in calling EMS. Greater attempts to learn about stroke were strongly associated with both stroke recognition and prompt calling of EMS. The rate of using Internet-related avenues to gain knowledge of stroke was 9.0%, which decreased with stroke risk score (20.9% in low-risk group vs. 6.5% in high-risk group) (See Supplementary Table S5, Additional file 1).

## Discussion

Our study shows that the knowledge of identifying stroke onset and intent to call EMS in Chinese CVD survivors were very poor and did not increase with recurrent stroke risk. Fortunately, CVD survivors were slightly more aware of the correct stroke response than the non-CVD population (OR: 1.11, 95% CI: 1.05–1.18) [18]. The rate of intent to call EMS (65.4%) [18] was almost similar to those previously reported in China [24] (58.5%), the United States [25] (62.9%), and Japan [26] (81.2%), but was significantly higher than the actual EMS usage rate in China (12.5–23.1%) [6, 8, 9, 27]. However, even in CVD survivors with high stroke risk score, one-third of them would not call EMS at the onset of a recurrent stroke.

Recurrent stroke is usually more devastating to the patient and is associated with higher treatment costs than the first episode [10, 11, 13]. A high prevalence of cardiovascular risk factors among CVD survivors was reported in our study, which accounted for most cases of stroke onset [28]. Multiple risk factors indicated a higher risk of recurrent stroke [28], which seems to be even higher in China due to poor control of these factors [29–32]. Unfortunately, in our study, higher recurrent stroke risk did not correlate with the intent to call EMS, which may delay access to reperfusion therapy [6, 9, 33]. CVD survivors, even those with high recurrent stroke risk, failed to regard underlying diseases as risk factors of stroke [17, 34, 35], and did not fully understand the danger of cardiovascular risk factors, and were unaware of their situation [17], probably due to poor education on secondary stroke prevention. For those with higher recurrent stroke risk, more intensive education program on risk factors, recurrent stroke recognition, and calling EMS should be implemented.

In contrast to results from the entire population, the odds of calling EMS among the CVD survivors depended only on a few factors, such as the region and annual income [18]. Moreover, contrary to the results from previous studies [18, 36], even highly educated CVD survivors did not perform better at recognizing stroke onset and calling EMS. Therefore, there are underlying factors other than lack of stroke education that remain unclear and require further study. This emphasizes the need to revise our previous programs to educate CVD survivors differently from non-CVD population and optimize secondary preventive programs on the appropriate response to stroke onset. Moreover, the limited number of associated factors that affect the odds of calling EMS among CVD survivors imply that targeted stroke-education programs can be more efficiently designed and conducted for CVD survivors than for the non-CVD population [18].

Low education, low income, and rural location were common among CVD survivors [37], which indicated a lower equity of needs and resource allocation to EMS [38, 39] and a poor capacity to curb the stroke burden [40, 41]. Additionally, our study demonstrated that only having ≥4 avenues for gaining knowledge on stroke was associated with calling EMS, indicating that the CVD survivors were not sensitive to solitary education campaigns, while multiple avenues showed synergistic effects [26]. Unfortunately, nearly half of CVD survivors with high recurrent risk had only one avenue to learn about stroke. With the availability of point-to-point functions, Internet-related avenues appear to be more appropriate to educate specific groups, but the Internet usage rate was extremely low among Chinese CVD survivors [26, 35, 42]. Finally, since CVD survivors face extremely high prevalence of risk factors, it is better to make them recognize that good control of these modifiable risk factors can reduce stroke risk [28], and that timely and proper response to stroke onset is critical to improving the outcome [36]. Thus, we should consider these unique and unfavorable points when launching education programs for Chinese CVD survivors.

Although ESRS predicts recurrent stroke risk, it could not be applied to our study directly [22]. Our model was partially different from ESRS, which might incur bias in predicting recurrent stroke risk. But, we found that prevalence of modifiable risk factors increased with stroke risk score, which indicated that CVD survivors with high stroke risk score might have highest odds to recurrent stroke [28].

The multistage nonrandom sampling design of this study and the selection from CNSSS was biased, although screening sites in urban and rural areas were selected in a 1:1 ratio [19]. Moreover, the prediction of recurrent stroke risk is complex and includes extensive risk factors. However, we only selected several factors to assess the risk for recurrent stroke. Therefore, the influence of other factors remained uninvestigated, such as the stenosis of intracranial artery [43]. Finally, further study is needed for the effect of stroke risk on patients’ response to stroke onset, which could optimize our strategy for stroke education.

## Conclusions

Our study found that stroke recognition and intent to call EMS in response to stroke-related symptoms were low among Chinese CVD survivors, and did not improve with stroke risk. CVD survivors had various recurrent stroke risks, but showed similar knowledge of stroke. Enhanced stroke education on stroke recognition and proper response should be made according to recurrent stroke risk of CVD survivors.

## Supplementary information

**Additional file 1.**

## Data Availability

The data sets in this study are available from the corresponding author on reasonable request.

## Abbreviations

AF: Atrial fibrillation
AIS: Acute ischemic stroke
BMI: Body mass index
CAR: Correct action rate
CVD: Cerebrovascular disease
CNSSS: China National Stroke Screening Survey
CI: Confidence interval
EMS: Emergency medical services
ESRS: Essen Stroke Risk Score
OR: Odds ratio
SRR: Stroke recognition rate
