# Supplementary material for: Increased recurrent risk did not improve cerebrovascular disease survivors’ response to stroke in China: a cross-sectional, community-based study
Source: BMC Neurol. 2020 Apr 21;20:147. doi: 10.1186/s12883-020-01724-1 (PMC7171759; doi:10.1186/s12883-020-01724-1)
Supplement: Supplementary file 1 — Additional file 1. [file 12883_2020_1724_MOESM1_ESM.docx]

**SUPPLEMENTAL MATERIAL**

**List of the FAST-RIGHT Investigators and Coordinators**

Bin Peng, MD (PI), Shengde Li, MD, Li-Ying Cui, MD, Nan Jiang, MD, Yuehui Hong, MD, Peking Union Medical College Hospital, Department of Neurology, Beijing; Longde Wang, MD, Stroke Control Project Committee, The National Health Commission, Beijing; Craig Anderson, MD, PhD, Neurological and Mental Health Division, The George Institute for Global Health, Faculty of Medicine, University of New South Wales, Sydney, Australia; The George Institute for Global Health, Peking University Health Science Center, Beijing, China; Chengdong Yu, MD, Guangliang Shan, MD, Chinese Academy of Medical Sciences, Institute of Basic Medical Sciences, Department of Epidemiology and Statistics, Beijing; Weidong Liu, MD, Liaocheng People’s Hospital, Neurosurgical Department, Liaocheng, Shandong; Jian Li, MD, Affliated Hospital of Weifang Medical University, Neurology Department, Weifang, Shandong; Chunpeng Gao, MD, Dalian Municipal Central Hospital, Disease Control and Prevention Office, Dalian, Liaoning; Suiqiang Zhu, MD, PhD, Huazhong University of Science and Technology, Tongji Hospital of Tongji Medical College, Department of Neurology, Wuhan, Hubei; Ping Xu, MD, Changde First People’s Hospital, Department of Neurology, Changde, Hunan; Tiemin Wei, MD, the Central Hospital of Lishui, Department of Cardiology, Lishui, Zhejiang; Yun Luo, MD, First People’s Hospital of Jiujiang, Department of Cardiovascular, Jiujiang, Jiangxi; Shengli Chen, MD, Chongqing Three Gorges Central Hospital, Department of Neurology, Chongqing; Dan Liu, MD, Jingmen First People’s Hospital, Jingmen, Hubei; Dongmei Xie, MD, Liuzhou Worker’s Hospital, Liuzhou, Guangxi; Dong Xu, Ningxia People’s Hospital, Yinchuan, Ningxia; Fei Wei, MD, Yichang Center People’s Hospital, Yichang, Hubei; Guanghui Wu, MD, Ningde City Hospital, Ningde, Fujian; Hongyan Li, MD, The People’s Hospital of Xinjiang Uygur Autonomous Region, Urumqi, Xinjiang; Hua Luo, MD, Affliated Hospital of Southwest Medical University, Luzhou, Sichuan; Jie Min, MD, The First People’s Hospital of Jingzhou, Jingzhou, Hubei; Jinhai Tang, MD, Jiangsu Province Hospital, Nanjing, Jiangsu; Jun Sun, MD, Wenzhou Central Hospital, Wenzhou, Zhejiang; Luoqing Li, MD, The First People Hospital of Yueyang, Yueyang, Hunan; Qi Yao, MD, Xinyu People’s Hospital, Xinyu, Jiangxi; Shilin Liu, MD, Pingxiang People’s Hospital, Pingxiang, Jiangxi; Wei Shi, MD, Affliated Hospital of Nantong University, Nantong, Jiangsu; Wei Yan, MD, The First People’s Hospital of Kashgar, Kashgar, Xinjiang; Xiaofei Yu, MD, Shuguang Hospital Affliated to Shanghai University of Traditional Chinese Medicine, Shanghai; Xiaopeng Luo, MD, Zhuzhou Central Hospital, Zhuzhou, Hunan; Xiaoxiang Peng, MD, Third people’s Hospital of Hubei Province, Wuhan, Hubei; Ya Zhang, MD, Dali Bai Autonomous Prefecture People’s Hospital, Dali, Yunnan; Yang Gao, MD, Yancheng City First People’s Hospital, Yancheng, Jiangsu; Ye Peng, MD, Harrison International Peace Hostipal, Hengshui, Hebei; Yongling Xue, MD, Qujing First People’s Hospital, Qujing, Yunnan; Zhi Lin, MD, Central People’s Hospital Of ZhanJiang, Zhanjiang, Guangdong; on behalf of the FAST-RIGHT study group.

Appendix1. The structure of FAST-RIGHT questionnaire with additional 4 questions about stroke awareness^1^

1. **FAST-RIGHT**
2. Which disease do the following symptoms probably mean? (Facial droop, Arm weakness, Speech disturbance)

- Correct (Participant should give an answer like “stroke”, “cerebral infarct”, “Cerebral thrombosis”, “ cerebral hemorrhage”, “ cerebral embolism”, “ apoplexy”)
- Incorrect（none of above）

1. What will you do when encountering above-mentioned symptoms? (Single selection)

- Self-observation at home (incorrect)
- Call the family, wait for them, then go to hospital (incorrect)
- Call emergency phone immediately (correct)

1. Have your relatives or colleagues suffered from these diseases?:Yes/No
2. How do you get such information? (Single selection/multiple Choice)

Newspaper/TV/Broadcast/Wechat/Internet/popular science and technology (PST) activities

1. **The other relative factors in this survey: brief introduction**

- Basic Information
- Demographic information (age, sex, education, family member, marriage, career, living status, annual income, medical insurance)
- Address (province)
- The status of conducting questionnaire (by self /others/phone)
- Lifestyle
- Smoking (Yes/No/Quit/Years)
- Drinking (Yes/No/Quit/Years)
- Exercise habit (often/less frequency)
- Dietary habits
- Family history (stroke, coronary artery disease [CHD], hypertension, diabetes mellitus, dyslipidemia]
- Diseases and management during survey (2015-2017)
- cerebrovascular disease [new stroke, types, Outpatient/Inpatient, mRS ]
- Coronary Heart Disease [new CHD, atrial fibrillation [AF]/drugs]
- Hypertension [drug adherence, monitoring frequency, qualification rate]
- Diabetes Mellitus [drug adherence, monitoring frequency, qualification rate]
- Dyslipidemia [drug adherence, monitoring frequency, qualification rate]
- Physical examination (body mass index [BMI], blood pressure [BP], cardiac auscultation)
- Grade of stroke risk

Hypertension Diabetes mellitus Dyslipidemia AF

BMI-Obesity Smoking Lack of exercise

Family history History of stroke History of transient ischemic attack (TIA)

Five levels: Stroke TIA High-Risk Moderate-Risk Low-Risk

- EEG, serum test (glucose, lipid, homocysteine), carotid artery ultrasound for new and prior high-risk, stroke and TIA group.
- Surgery and interventional therapy during this survey
- Carotid artery [CAS, CEA, Extracranial-Intracranial Bypass]
- Coronary artery [PCI, CABG]
- Surgery/Interventional therapy for Intracranial hemorrhage [Yes/No]

Appendix 2. Definition of Risk Factors for Stroke^1^

| **Risk factor** | **Criteria** |
| --- | --- |
| Smoking | ≥6 months in life (accumulative or consecutive) |
| Alcohol drinking | Drinking habit without heavy drinking  OR  ≥100mL spirit alcohol ≥ 3 times per week (self report) |
| Hypertension | 1. Systolic BP ≥140mmHg or diastolic BP ≥90mmHg, or taking BP-lowering drugs   OR   1. ABPM: 24 h mean BP ≥130/80 mmHg, or daytime BP ≥135/85mmHg, or nocturnal BP ≥120/70mmHg   OR  3. Home BP monitoring ≥135/85 mmHg(self report) |
| Dyslipidemia | LDL-C ≥1.8mmol/L, or HDL-C <1.04mmol/L, or TC ≥6.22mmol/L, or TG≥2.26mmol/L |
| Diabetes | 1. Fasting glucose ≥7.0 mmol/L or non-fasting blood glucose ≥11.0mmol/L with diabetes mellitus symptoms   OR  2. Fasting glucose ≥7.0mmol/L or non-fasting blood glucose ≥11.0mmol/L more than two times without typical DM symptoms  OR  3.OGTT (75g glucose): 2 h blood glucose ≥11.0 mmol/L  OR  4.Taking glucose-lowering drugs |
| Significant overweight or obese | BMI ≥26.0 kg/m^2^ |
| AF | Either a history of persistent AF or supported by past ECG or ECG examination in this survey and confirmed by a cardiologist |
| Physical exercise | 1. ≥ 30 min of medium strength and above exercise every time, >3 times per week   OR  2. Moderate or heavy manual workers |
| Family history of stroke | Any parent or sibling with stroke; further inquiry and confirmation by a neurologist |
| History of stroke. | Either neurological deficit symptom at onset or symptomatic lacunar cerebral infarction on imaging, confirmed by a neurologist |
| TIA | Sudden onset focal/global neurological deficit lasting less than 24h, usually alleviating within 30minutes, excluding non-angiogenesis, and confirmed by a neurologist |

Appendix 3.Stroke risk score based on Essen Stroke Risk Score

| **Risk factors** | **Score** |
| --- | --- |
| **Age** |  |
| < 65 | 0 |
| 65-75 | 1 |
| >75^a^ | 2 |
| **Hypertension** | 1 |
| **Diabetes Mellitus** | 1 |
| **Heart disease^b^** | 2 |
| **Smoking^c^** | 1 |

The score range is 0 to 7.

^a^ 75-99

^b^ Heart disease includes atrial fibrillation (AF), valvular heart disease, coronary heart disease and other heart disease.

^c^ Smoking includes current, former and passive smoking.

Table S1. Missing data for each variable about cerebrovascular disease survivors

| Subgroup | N |
| --- | --- |
| Age^a^ | 0 |
| Sex | 0 |
| Site | 0 |
| Region | 0 |
| BMI^b^ | 7 |
| Education | 0 |
| Personal annual income | 3 |
| Living status | 3 |
| Children number | 5 |
| Stroke amongst people around them | 0 |
| Number of avenues taken to participate | 0 |
| Smoking status | 0 |
| Level of exercise | 0 |
| Drink | 0 |
| Family history of stroke | 4 |
| History of heart disease | 0 |
| History of hypertension | 1 |
| History of diabetes | 3 |
| History of dyslipidemia | 2 |
| Stroke risk score | 3 |

^a^Age ≥100 was classified as missing

^b^BMI >50 or <10 was classified as missing

**Table S2. SRR and CAR by stroke risk score in CVD survivors**

|  | SRR | |  | CAR | |
| --- | --- | --- | --- | --- | --- |
|  | n/N (%, 95%CI) | p value |  | n/N (%, 95%CI) | p value |
| Stroke risk score ^a^ |  | 0.0014 |  |  | 0.004 |
| 0 | 503/565 (89.0, 86.5-91.6) |  |  | 377/565 (66.7, 62.8-70.6) |  |
| 1–3 | 3841/4509 (85.2, 84.2-86.2) |  |  | 2897/4509 (64.3, 62.9-65.7) |  |
| 4–7 | 1001/1213 (82.5, 80.4-84.7) |  |  | 840/1213 (69.3, 66.7-71.9) |  |

SRR: Stroke recognition rate; CAR: correct action rate.

N: total in each cell; n: number for recognizing stroke/correct action to stroke.

^a^ The score range was 0 to 7.

**Table S3. Relation of recognition of stroke and correct action after stroke**

|  | Correct action  n/N (%) | Incorrect action  n/N (%) |
| --- | --- | --- |
| Recognition of stroke | 3633/5348 (67.9) | 1715/5348 (32.1) |
| Unknown of stroke | 482/942 (51.2) | 460/942 (48.8) |

**Table S4. Comparisons of socio-demographic factors in 0, 1-3, and 4-7 stroke risk score**

|  | Stroke risk score，n (%) | | |  |
| --- | --- | --- | --- | --- |
|  | 0 | 1-3 | 4-7 | P value |
| Age (years) |  |  |  | <0.0001 |
| 40–49 | 104 (18.4) | 189 (4.2) | 8 (0.7) |  |
| 50–59 | 273 (48.3) | 712 (15.8) | 54 (4.5) |  |
| 60–69 | 188 (33.3) | 1865 (41.4) | 320 (26.4) |  |
| 70–79 | 0 (0) | 1364 (30.3) | 579 (47.7) |  |
| 80–99 | 0 (0) | 379 (8.4) | 252 (20.8) |  |
| Sex |  |  |  | <0.0001 |
| Male | 170 (30.1) | 2160 (47.9) | 605 (49.9) |  |
| Female | 395 (69.9) | 2349 (52.1) | 608 (50.1) |  |
| Site |  |  |  | <0.0001 |
| Urban | 201 (35.6) | 1990 (44.1) | 738 (60.8) |  |
| Rural | 364 (64.4) | 2519 (55.9) | 475 (39.2) |  |
| Regions |  |  |  | <0.0001 |
| North + Northeast | 55 (9.7) | 461 (10.2) | 202 (16.7) |  |
| East | 130 (23.0) | 1088 (24.1) | 292 (24.1) |  |
| Central | 249 (44.1) | 1990 (44.1) | 487 (40.1) |  |
| South | 26 (4.6) | 309 (6.9) | 56 (4.6) |  |
| Southwest | 67 (11.9) | 461 (10.2) | 94 (7.7) |  |
| Northwest | 38 (6.7) | 200 (4.4) | 82 (6.8) |  |
| Education |  |  |  | <0.0001 |
| ≤ Primary | 231 (40.9) | 2411 (53.5) | 605 (49.9) |  |
| Middle/High school | 304 (53.8) | 1887 (41.85) | 539 (44.4) |  |
| ≥ College | 30 (5.3) | 211 (4.7) | 69 (5.7) |  |
| Personal Annual Income (US $) |  |  |  | <0.0001 |
| < 731 | 242 (42.8) | 1832 (40.7) | 405 (33.4) |  |
| 731-2923 | 199 (35.2) | 1470 (32.6) | 334 (27.5) |  |
| > 2923 | 124 (30.0) | 1204 (26.7) | 474 (39.1) |  |
| Living Status^a^ |  |  |  | <0.0001 |
| With family | 548 (97.0) | 4214 (93.5) | 1084 (89.4) |  |
| With others | 17 (3.0) | 292 (6.5) | 129 (10.6) |  |
| Children number |  |  |  | <0.0001 |
| 0 | 2 (0.4) | 43 (1.0) | 12 (1.0) |  |
| 1 | 171 (30.3) | 866 (19.2) | 190 (15.7) |  |
| 2–3 | 348 (61.7) | 2797 (62.1) | 730 (60.2) |  |
| ≥ 4 | 43 (7.6) | 799 (17.7) | 281 (23.2) |  |
| Avenues^b^ |  |  |  | 0.0019 |
| 1 | 263 (46.6) | 2254 (50.0) | 585 (48.2) |  |
| 2–3 | 262 (46.4) | 2090 (46.4) | 582 (48.0) |  |
| 4–6 | 40 (7.1) | 165 (3.7) | 46 (3.8) |  |
| Family history of stroke |  |  |  | <0.0001 |
| No | 422 (74.7) | 3201 (71.0) | 760 (62.7) |  |
| Yes | 108 (19.1) | 1193 (26.5) | 407 (33.6) |  |
| Unknown | 35 (6.2) | 111 (2.5) | 46 (3.8) |  |

^a^ With family includes living with spouse/children; With others includes being single, living in a nursing home, and with other people

^b^ The number of avenues taken to learn about stroke

**Table S5. Avenues to learning about acute stroke**

|  |  | Stroke risk score | | | |
| --- | --- | --- | --- | --- | --- |
|  | CVD  n/N (%) | 0  n/N (%) | 1-3  n/N (%) | 4-7  n/N (%) | P value |
| Newspaper | 1597/6290 (25.4) | 149/565 (26.4) | 1095/4509 (24.3) | 351/1213 (28.9) | 0.0036 |
| TV | 4825/6290 (76.7) | 425/565 (75.2) | 3453/4509 (76.6) | 944/1213 (77.8) | 0.4529 |
| Broadcast | 1156/6290 (18.4) | 121/565 (21.4) | 817/4509 (18.1) | 216/1213 (17.8) | 0.1393 |
| Wechat | 302/6290 (4.8) | 67/565 (11.9) | 196/4509 (4.4) | 39/1213 (3.2) | <0.0001 |
| Internet | 267/6290 (4.2) | 51/565 (9.0) | 176/4509 (3.9) | 40/1213 (3.3) | <0.0001 |
| PST | 2901/6290 (46.1) | 245/565 (43.4) | 2109/4509 (46.8) | 547/1213 (45.1) | 0.2216 |
| PST-only^a^ | 1006/6290 (16.0) | 76/565 (13.5) | 746/4509 (16.5) | 184/1213 (19.3) | 0.1137 |

PST denotes popular science and technology.

^a^ Part of participants only selecting PST activities included those who reported no avenue to learn stroke. They were regarded as having a PST activities pathway according to accepting information about stroke in the previous China National Stroke Screening Survey. The real number without any pathways is unknown.

Figure S1. Data preparation and cleaning process^1^

**Non-CVD group:** 18 1240

**CVD survivors**: 6290

Delete: Missing data: 93

Delete: 2437

**Raw data in primary survey (N=243 279)**

Delete: 51126

**Data according to age standard (N=240 842)**

**Data of finishing face-to-face interview by oneself (N=189 716)**

**Final dataset (N=187 723)**

Delete: 1993

Step7: Did not answer question of stroke recognition (n= 1991)

Step8: Did not answer question of response to stroke (n= 2)

Step1: Death (n= 2413)

Step2: Test population^a^ (n=5)

Step3: Age < 40 years (n=13) and age ≥ 146 year (n=6)

Step4: Loss of follow-up (n= 6338)

Step5: Finish interview by others (n= 7240)

Step6: Finish interview via phone^b^ (n= 37548)

^a^ Virtual population to test the data management system.

^b^ To ensure the accuracy, integrity, and creditability, residents finishing questionnaires via phone in CNSSS was not included in FAST-RIGHT study.

Reference

1. Li S, Cui LY, Anderson C, Zhu S, Xu P, Wei T, et al. Public awareness of stroke and the appropriate responses in China: a cross-sectional community-based study (FAST-RIGHT). Stroke. 2019;50:455–62. https://doi. org/10.1161/STROKEAHA.118.023317.
